# Nutritional quality of regular and pureed menus in Canadian long term care homes: an analysis of the Making the Most of Mealtimes (M3) project

**DOI:** 10.1186/s40795-017-0198-3

**Published:** 2017-10-23

**Authors:** Vanessa Vucea, Heather H. Keller, Jill M. Morrison, Alison M. Duncan, Lisa M. Duizer, Natalie Carrier, Christina O. Lengyel, Susan E. Slaughter

**Affiliations:** 1 Department of Kinesiology, University of Waterloo, 200 University Ave W, Waterloo, ON N2L 3G1 Canada; 2 Schlegel-University of Waterloo Research Institute for Aging, 250 Laurelwood Drive, Waterloo, ON N2J 0E2 Canada; 3 Department of Human Health and Nutritional Sciences, University of Guelph, Guelph, ON N1G 2W1 Canada; 4 Department of Food Science, University of Guelph, Guelph, ON N1G 2W1 Canada; 5 École des sciences des aliments, de nutrition et d’études familiales, Faculté des sciences de la santé et des services communautaires, Université de Moncton, Moncton, NB E1A 3E9 Canada; 6 Department of Human Nutritional Sciences, University of Manitoba, Winnipeg, MB R3T 2N2 Canada; 7 Faculty of Nursing, University of Alberta, Edmonton, AB T6G 1C9 Canada

**Keywords:** Nutrients, Long term care, Older adults, Dietary reference intake, Menu quality, Pureed texture

## Abstract

**Background:**

Long term care (LTC) menus need to contain sufficient nutrients for health and pureed menus may have lower nutritional quality than regular texture menus due to processes (e.g., recipe alterations) required to modify textures. The aims of this study were to: determine adequacy of planned menus when compared to the Dietary Reference Intake (DRI); compare the energy, macronutrients, micronutrients and fibre of pureed texture and regular texture menus across LTC homes to determine any texture, home or regional level differences; and identify home characteristics associated with energy and protein differences in pureed and regular menus.

**Methods:**

Making the Most of Mealtimes (M3) is a cross-sectional multi-site study that collected data from 32 LTC homes in four Canadian provinces. This secondary analysis focused on nutrient analysis of pureed and regular texture menus for the first week of the menu cycle. A site survey captured characteristics and services of each facility, and key aspects of menu planning and food production. Bivariate analyses were used to compare menus, within a home and among and within provinces, as well as to determine if home characteristics were associated with energy and protein provision for both menus. Each menu was qualitatively compared to the DRI standards for individuals 70+ years to determine nutritional quality.

**Results:**

There were significant provincial and menu texture interactions for energy, protein, carbohydrates, fibre, and 11 of 22 micronutrients analyzed (*p* < 0.01). Alberta and New Brunswick had lower nutrient contents for both menu textures as compared to Manitoba and Ontario. Within each province some homes had significantly lower nutrient content for pureed menus (*p* < 0.01), while others did not. Fibre and nine micronutrients were below DRI recommendations for both menu textures within all four provinces; variation in nutritional quality existed among homes within each province. Several home characteristics (e.g., for-profit status) were significantly associated with higher energy and protein content of menus (*p* < 0.01).

**Conclusions:**

There was variability in nutritional quality of menus from LTC homes in the M3 sample. Pureed menus tended to contain lower amounts of nutrients than regular texture menus and both menus did not meet DRI recommendations for select nutrients. This study demonstrates the need for improved menu planning protocols to ensure planned diets meet nutrient requirements regardless of texture.

**Trial registration:**

ClinicalTrials.gov ID: NCT02800291, retrospectively registered June 7, 2016.

**Electronic supplementary material:**

The online version of this article (10.1186/s40795-017-0198-3) contains supplementary material, which is available to authorized users.

## Background

Menu planning in long term care (LTC) homes is an essential activity to support the nutritional needs of older adults who are often nutritionally compromised [1–4]. Menu planning in this context needs to consider the characteristics of residents in the facility (i.e., needs and preferences), the practicalities of food production, and the nutritional content of the provided food (i.e., variety, quality, and balance) to promote health, food intake, nutritional status, and quality of life [1, 5]. This paper will focus on the nutritional quality of menus as planned in LTC. Canadian LTC homes are governed by provincial regulations (e.g., availability of services, level of public funding) that can influence the content of menus. For example, Ontario requires a per capita minimum amount of funding per day for food [6], while other provinces do not. Further, Ontario bases their menu planning on the Dietary Reference Intake and the *Eating well with Canada’s Food Guide (CFG)* [6–10], yet Manitoba, Alberta, and New Brunswick only use the CFG [11–13]. Planned menus can be prepared by dietitians and/or nutrition managers, but it is specified in the Ontario legislation that dietitians must sign off on the menu meeting the DRI [6, 10]. Thus with this variation in menu planning regulations, it is not surprising that planned menus have been previously found to be insufficient in protein, energy, and micronutrients [14–17]. Expertise and effort put into planning menus including obtaining input of residents and family councils, and the challenges of using CFG as the basis for menu planning due to the portion sizes being too large for residents also lead to variability [1, 10, 16, 17].

Deficits in menu quality may be exacerbated for texture modified menus, especially pureed menus [14, 16–21] which have been shown to contain fewer calories and offer less nutritional quality as compared to unmodified texture food (i.e., regular texture food) [14–16]. Currently, there are no guidelines for preparing in-house MTFs [22, 23]. This has many implications such as inconsistent use and confusion around terminology for MTF as well as food particle size, variation in nutritional and sensory quality, and safety [21–23]. Furthermore, in-house pureed products tend to be altered from the regular recipe that may or may not be standardized, thus it is hard to obtain the accurate nutritional content of these foods [3, 24, 25]. This has led to some LTC homes choosing commercial rather than in-house production of MTF. Considerations for using commercial products, especially for pureed food, is dependent on: menu requirements such as variety, choice, food groups, and comparable menu items between regular texture food and MTF; cost as commercial products tend to be more expensive; capacity for commercial bulk purchasing which will vary with the number of residents requiring MTF; acceptability and palatability, since some food can be challenging to modify in-house; and standardization to ensure consistency and quality in a product which may be more challenging to provide in-house (e.g., pureed meat products) [22, 26]. In addition to menu planning the availability and use of commercially prepared modified textured food (MTF) may also influence nutrient quality of products. With the development and use of more commercially prepared pureed foods, it is not known if these menus are now on par with regular texture menus, especially as variability in nutritional quality of commercial products has also been shown [26, 27].

Research to date demonstrates potential variability and deficits in terms of nutrient content of regular and pureed texture menus, but is limited in the number and regional diversity of LTC homes included in the sample, number of menu days assessed, and contrasting textures. Further, understanding why some of these differences exist with respect to LTC home characteristics requires investigation and is currently unknown. The Making the Most of Mealtimes (M3) study, conducted in 32 LTC homes in four provinces of Canada, has the opportunity to address this evidence gap. This study was designed to answer the following research questions: 1) Is the pureed diet provided as planned for one week significantly different in energy, macronutrients (i.e., protein and carbohydrates), micronutrients, and fibre as compared to the regular texture diet from that home; 2) How do the regular and pureed texture menus compare to the DRIs; 3) Are there regional (i.e., by province) and LTC home differences; and 4) What LTC home characteristics are associated with higher energy and protein provision on the menu for regular texture and pureed texture?

## Methods

### Study sample and design

M3 was a cross-sectional multi-site Canadian study; details on the primary research questions and comprehensive data collection can be found in the protocol [28]. This was a secondary study, based solely on the planned menus of the homes. A total of 32 LTC homes were purposively recruited across four Canadian provinces (8 homes per province) in Canada: Alberta (AB), Manitoba (MB), New Brunswick (NB), and Ontario (ON), [27]. Eligibility criteria for LTC homes included the following: 1) in operation for at least six months; and 2) having a minimum of 50 residents who met the resident eligibility criteria. For-profit and not-for profit LTC homes were recruited and diversity in the sample was also attempted by including those with special characteristics such as a high proportion of individuals who were cultural minorities, independent operators and large corporations, rural or urban settings and faith-based LTC homes.

### Data collection measures

Each home provided a complete menu for analysis of regular and pureed textures. A site survey was also completed by each LTC home, which captured characteristics of each facility (e.g., profit status), key aspects of menu planning and food production (e.g., menu cycle length), and other pertinent services in the LTC home (e.g., dietitian time). This survey was typically completed by the director of food services and other key personnel (e.g., dietitian). Variables used in this analysis and collected from the site survey included: home sector, age of home, number of beds, menu cycle length, date of last full revision of the menu, guidelines for menu planning, dietitian time, per capita raw food cost, methods of food preparation used, the proportion of commercial versus in-house production of regular and modified texture foods, production of modified texture foods, and recipe availability for modified texture foods.

#### Nutrient analysis

Energy and macro- and micronutrient analysis of the provided regular and pureed texture menus was completed for each LTC home concurrently during data collection for the M3 study. Eight highly trained research assistants (two per province), who were involved in direct food weighing and assessing of meals for the M3 study, also analyzed these menus. Unlike a typical external researcher, they had intimate knowledge of the menu items from seeing them served to residents and had the opportunity to clarify any questions on menu content with LTC home staff. The analysis was conducted for the first week of the menu cycle for first choice food products; for this analysis snacks were excluded to promote consistency, as less than half (*n* = 13) of the homes included snacks in the planned menu. Prior work, suggests that a single week is sufficient to demonstrate differences where they exist among homes [18]. To accurately analyze the menu, home recipes, specific food product labels, and standard portion sizes were identified for each menu item and incorporated into the analysis; where necessary the research assistants consulted with cooks or dietary food service supervisors to clarify items. The two research assistants in each LTC home worked in tandem, checking each other’s nutrient analysis of recipe items. Nutrient analysis was completed using ESHA Food Processor software (Version 10.14.1) using the Canadian Nutrient File [29], where appropriate, for foods that varied in fortification practices with the United States Department of Agriculture Nutrient File [30]. A code-book was created per province to promote consistency in database choices for selected menu items [28]. A random check of menu items was completed by the first author to ensure accuracy in use of the Canadian Nutrient File for key foods among provinces; this check was especially focused for provinces that showed significant nutrient differences from others to confirm that differences in nutrient content were not a result of a provincial research assistant bias in recipe/food item selection from the database. For the analysis of food and fluid items in the first menu week, the energy and nutrients (macronutrients and micronutrients) per day for each texture (i.e., pureed and regular) were determined and then averaged for the week to determine the average nutritional quality per day of the planned menu.

### Statistical analysis

Data were analyzed using Statistical Analysis System software for Windows (Version 9.4). First, descriptive statistics were used to compute means for continuous home-level variables and frequencies for the categorical home-level variables. Additionally, descriptive statistics were used to compute means for numerical diet variables by LTC home (i.e. energy, macronutrients, micronutrients, and fibre), where means were expressed per day per menu texture. An average of these variables per week for each menu texture was also created per LTC home. To determine the LTC home characteristics associated with energy and protein provision for pureed and regular texture menus, a Student’s t-test was used to compare intake to categorical variables with two levels. A one-way analysis of variance (ANOVA) was used for variables with more than two levels and a Pearson’s correlation test was performed for continuous variables. A Student’s t-test was used to compare the average nutrient content of the planned pureed menu to the regular texture menu in each LTC home. A two-way ANOVA was used to compare menus among LTC homes within each province for each menu texture. Next, an average pureed week and regular week was created for the entire province, allowing for comparisons using a two-way ANOVA to determine any differences among provinces in pureed and regular texture menus overall. The DRI for those 70+ years was used for comparisons. The recommended dietary allowance (RDA) or adequate intake (AI) for males was used for comparisons so it would encompass recommended levels for both genders, since levels for some nutrients were higher for males. To adjust for a type one error due to multiple tests conducted within home and among province, statistical significance was set at α = 0.01 for comparisons.

## Results

### Characteristics of the long term care homes

Table 1 summarizes characteristics of the LTC homes for the four provinces in the M3 study. Of the LTC homes sampled among the provinces, Alberta, Manitoba, and Ontario had larger facilities as compared to New Brunswick. Manitoba had the shortest menu cycle length (median = 3 weeks, range: 3–5 weeks), followed by Alberta (median = 4 weeks, range: 3–4 weeks), Ontario (median = 4 weeks, range: 3–5 weeks), and New Brunswick (median = 4 weeks, range: 3–6 weeks). Looking at the last full revision of the planned menu, seven of the eight LTC homes sampled in Ontario had revised their menu less than six months from the date of data collection, however Alberta only had four LTC homes that had revised their menu in this time frame. Ontario homes spent on average the most for raw food purchasing per resident, followed by Manitoba, Alberta, and New Brunswick. Manitoba had the highest proportion of commercially prepared food with no further preparation needed (mean = 30.0%, range: 20–100%). Alberta, New Brunswick, and Ontario had an average proportion between 10% and 15% of commercially prepared food with lower ranges as well.

**Table 1:** Characteristics of the LTC homes for each province in the M3 study

	Province			
	Alberta	Manitoba	New Brunswick	Ontario
Home Sector, % (n)				
For-profit	50.0 (4)	37.5 (3)	12.5 (1)	25.0 (2)
Non-profit	50.0 (4)	62.5 (5)	87.5 (7)	75.0 (6)
Age of home in years, mean (range)	28.6 (4–51)	35.4 (14–70)	34.9 (5–47)	25.9 (11–59)
Number of beds per LTC home, mean (range)	151.5 (100–226)	161.9 (57–233)	87.3 (50–200)	138.9 (84–238)
Menu cycle length in weeks, median (range)	4 (3–4)	3 (3–5)	4 (3–6)	4 (3–5)
Last full revision of the menu, % (n)				
Less than 6 months	50.0 (4)	75.0 (6)	75.0 (6)	87.5 (7)
6–12 months	37.5 (3)	25.0 (2)	12.5 (1)	12.5 (1)
13–18 months	12.5 (1)	0	0	0
More than 18 months	0	0	12.5 (1)	0
Menu planning based on Canada’s Food Guide (CFG), % (n)	100.0 (8)	100.0 (8)	100.0 (8)	100.0 (8)
Menu planning based on other guidelines^§^, % (n)	37.5 (3)	12.5 (1)	12.5 (1)	37.5 (3)
Dietitian time (hours per week), mean (range)	22.5 (15.0–37.5)	21.8 (7.5–38.8)	13.8 (0–37.5)	16.6 (7.0–35.0)
Raw food cost ($ per resident/day), mean (range) *	7.50 (6.10–8.3)	7.80 (6.20–9.00)	7.20 (6.30–7.90)	8.50 (7.30–12.50)
Methods of food preparation primarily used, % (n)				
Traditional/conventional system	62.5 (5)	50.0 (4)	62.5 (5)	100.0 (8)
Ready prepared system (bulk or individual reheat)	0	25.0 (2)	37.5 (3)	0
Both methods used ^a^	37.5 (3)	25.0 (2)	0	0
Proportion of food production is commercially prepared ^b^, median (range)	10.0 (2–70)	30.0 (20–100)	15.0 (2–60)	10.0 (0–30)
Production of modified texture foods in the home, % (n)				
Modified from regular texture in home	37.5 (3)	50.0 (4)	37.5 (3)	62.5 (5)
Purchased all modified texture food	12.5 (1)	12.5 (1)	25.0 (2)	0
Both methods used ^c^	50.0 (4)	37.5 (3)	37.5 (3)	37.5 (3)
Recipes are available for modified textures prepared from the regular texture, % (n) **				
Yes	25.0 (2)	37.5 (3)	25.0 (2)	75.0 (6)
Only some items	37.5 (3)	25.0 (2)	37.5 (3)	25.0 (2)
No	25.0 (2)	25.0 (2)	12.5 (1)	0

Descriptive statistics were used to compute means for continuous variables and frequencies for the categorical variables. Numerical and ordinal data are mean (standard deviation) and median where appropriate. Categorical and ordinal data are % (n). Total *n* = 32, where * *n* = 27 and ** *n* = 28

^§^Other guidelines for menu planning in addition to using Canada’s Food Guide include: LTC accommodation standards, LTC homes act, Dietitians of Canada menu planning, home specific guidelines

^a^Both methods used = traditional/conventional and ready prepared systems used

^b^Proportion of food production is commercially prepared with no further preparation needed

^c^Both methods used = modified from regular in-home & purchased

Table 2 provides the home characteristics associated with energy and protein provision for the 32 LTC homes; energy represents potential quantity of food while protein is a proxy for the quality of provided food. For-profit homes provided significantly higher amounts of energy and protein compared to non-profit homes for both menu textures (energy for regular: *t* = 5.3, *p* < 0.0001; pureed: *t* = 4.6, *p* < 0.0001) (protein for regular: *t* = 4.5, *p* < 0.0001; pureed: *t* = 3.6, *p* = 0.0005), while the number of beds was positively associated with energy provision from the regular texture menu (*r* = 0.2, *p* = 0.006). Menu cycle length was related to energy and protein provided by both menu textures in that a menu cycle of six weeks provided significantly lower protein and energy intake than three, four, and five week menu cycles (energy for regular: F(3, 220) = 8.2, *p* < 0.0001; pureed: F(3, 220) = 11.4, *p* < 0.0001) (protein for regular: F(3, 220) = 8.9, *p* < 0.0001; pureed: F(3, 220) = 12.3, *p* < 0.0001). Frequency of menu revision was associated with energy and protein provided by both menu textures among the 32 homes (energy for regular: F(3, 220) = 12.1, *p* < 0.0001; pureed: F(3, 220) = 16.2, *p* < 0.0001) (protein for regular: F(3, 220) = 6.2, *p* = 0.0004; pureed: F(3, 220) = 11.2, *p* < 0.0001) and a revision that occurred more than 18 months prior to the M3 data collection was associated with lower amounts of energy and protein for both menu textures. Raw food cost per resident was positively associated with energy provided by the pureed texture menus (*r* = 0.2, *p* = 0.001) and the proportion of commercial food use was positively associated with energy provision from the regular texture menus (r = 0.2, *p* = 0.005).

**Table 2:** Home characteristics associated with energy and protein provision from menus

		Energy						Protein					
		Regular Texture Menu			Pureed Texture Menu			Regular Texture Menu			Pureed Texture Menu		
		kcal per day	Test Statistic	*P*-value	kcal per day	Test Statistic	*P*-value	grams per day	Test Statistic	*P*-value	grams per day	Test Statistic	*P*-value
Home Sector, % (n)													
For-profit	31.2 (10)	2255.2 (381.2)	5.3 ^a^	<0.0001	2023.5 (422.0)	4.6 ^a^	<0.0001	95.3 (18.7)	4.5 ^a^	<0.0001	89.1 (16.2)	3.6 ^a^	0.0005
Non-profit	68.8 (22)	1968.9 (372.2)			1699.7 (511.5)			82.5 (20.4)			79.0 (25.6)		
Number of beds per LTC home, median (IQR)	126.0 (87.5–190.0)	2058.4 (397.1)	0.2 ^b^	0.006	1800.9 (507.2)	0.1 ^b^	0.44	86.5 (20.7)	0.2 ^b^	0.02^b^	82.2 (23.6)	0.1 ^b^	0.05
Menu cycle length in weeks, % (n)													
3 weeks^d^	40.6 (13)	2109.5 (419.0) ^g^	8.2 ^c^	<0.0001	1767.6 (484.2) ^f,g^	11.4 ^c^	<0.0001	84.0 (19.0) ^g^	8.9 ^c^	<0.0001	79.5 (20.2) ^f,g^	12.3 ^c^	<0.0001
4 weeks^e^	40.6 (13)	2035.9 (370.4) ^g^			1805.9 (404.8) ^f,g^			90.2 (22.2) ^g^			85.1 (21.8) ^g^		
5 weeks^f^	12.5 (4)	2187.6 (302.6) ^g^			2159.6 (678.5) ^d,e,g^			94.0 (16.3) ^g^			95.7 (30.5) ^g^		
6 weeks^g^	6.3 (2)	1613.5 (281.1) ^d,e,f^			1266.7 (326.2) ^d,e,f^			64.2 (11.5) ^d,e,f^			53.8 (9.1) ^d,e,f^		
Last full revision of the menu, % (n)													
< 6 months^h^	75.0 (24)	2088.5 (389.5) ^k^	12.1 ^c^	<0.0001	1776.3 (473.3) ^i,k^	16.2 ^c^	<0.0001	87.4 (21.2) ^k^	6.2 ^c^	0.0004	81.8 (22.1) ^k^	11.2 ^c^	<0.0001
6–12 months^i^	18.8 (6)	2111.9 (323.6) ^k^			2106.2 (477.2) ^h,j,k^			89.9 (17.2) ^k^			91.8 (24.6) ^k^		
13–18 months^j^	3.1 (1)	1790.8 (182.0)			1437.4 (200.0) ^i^			74.5 (11.3)			75.0 (13.3)		
> 18 months^k^	3.1 (1)	1282.1 (243.3) ^h,i^			921.1 (60.7) ^h,i^			57.5 (8.6) ^h,i^			40.6 (3.0) ^h,i^		
Dietitian time (hours per week), median (IQR)	17.0 (13.0–22.5)	2058.4 (397.1)	0.1 ^b^	0.07	1800.9 (507.2)	−0.1 ^b^	0.13	86.5 (20.7)	0.1 ^b^	0.43	82.2 (23.6)	−0.02 ^b^	0.79
Raw food cost ($ per resident/day), median (IQR) *	7.7 (7.1–8.3)	2067.0 (383.3)	0.1 ^b^	0.08	1817.0 (491.2)	0.2 ^b^	0.001	87.2 (20.7)	−0.1 ^b^	0.28	83.1 (23.4)	0.1 ^b^	0.41
Proportion of commercial food, median (IQR)	17.5 (10–30)	2058.4 (397.1)	0.2 ^b^	0.005	1800.9 (507.2)	0.04 ^b^	0.52	86.5 (20.7)	−0.1 ^b^	0.15	82.2 (23.6)	−0.1 ^b^	0.31

Statistical tests were performed to compare home characteristics with energy and protein provision from the regular and pureed texture menus among the 32 LTC homes from four provinces

Total n = 32, where * n = 27

*Abbreviations*: *kcal* kilocalorie, *IQR* interquartile range

^a^Statistical value is a t-value based on a Student’s t-test

^b^Statistical value is a Pearson correlation coefficient

^c^ANOVA was performed and statistical value is a F-value

^d, e, f, g, h, i, j, k^Values with different superscripts indicate a significant difference at *p* < 0.01

### Comparison of regular and pureed texture menus across provinces

Table 3 presents the average nutrient content of the regular and pureed texture menus for the 32 LTC homes from four Canadian provinces. On average, the amount of energy offered per day from the pureed menus was 1801 kcal (standard deviation (SD) = 507.2) and 2058 kcal (SD = 397.1) from the regular texture menus. A two-way ANOVA comparing province and menu texture for energy revealed significant main effects (province F(3, 440) = 81.7, *p* < 0.0001; menu texture F(1440) = 56.6, *p* < 0.0001), which were modified by a significant interaction between province and menu texture (F(3440) = 6.7, *p* = 0.0002). The presence of a significant interaction means that differences in energy provision by province was not consistent by texture menu type; in some provinces pureed menus had higher energy, while in others regular menus had the higher value. For protein, the pureed menus contained on average 82.2 g (SD = 23.6) per day while the regular texture menu had 86.5 g (SD = 20.7). The amount of protein offered daily in both of the menu textures exceeded the RDA of 56 g per day for those 70+ years. Significant provincial and menu texture main effect and interactions were noted for protein, carbohydrate and fibre, meaning that consistent with energy, there were provincial and texture differences, but these were not consistent. Both menu textures met carbohydrate (130 g/d) but not fibre (30 g/d) recommendations. Among the four provinces, New Brunswick and Alberta had lower averages for all three macronutrients for both menu textures. New Brunswick had the lowest average of fibre for both menu textures compared to the other three provinces.

**Table 3:** Comparison of menus as planned among the 32 LTC homes from four provinces

Among All Provinces			
	RDA (70+ M)	Regular Texture Menu	Pureed Texture Menu
	AI* (70+ M)	Mean (SD)	Mean (SD)
Energy (kcal)	n/a	2058.4 (397.1)^a^	1800.9 (507.2)
Protein (g)	56	86.5 (20.7)^a^	82.2 (23.6)
Carbohydrates (g)	130	265.8 (52.2)^a^	229.2 (66.9)
Fibre (g)	**30**	**20.9 (5.05)** ^**a**^	**16.9 (5.68)**
Vitamin A (RAE)	900	1061.8 (618.0)^b^	982.4 (503.9)
Vitamin B1 (mg)	1.2	1.67 (0.50)^a^	1.38 (0.60)
Vitamin B2 (mg)	1.3	2.43 (1.03)^b^	2.28 (0.94)
Vitamin B3-NE (mg)	16	34.8 (9.85)^a^	28.8 (12.3)
Vitamin B6 (mg)	**1.7**	**1.68 (0.52)** ^**a**^	**1.48 (0.62)**
Vitamin B12 (mcg)	2.4	5.55 (4.89)	5.13 (3.04)
Vitamin C (mg)	90	130.6 (74.0)^b^	128.6 (69.1)
Vitamin D (mcg)	**20**	**7.52 (3.78)** ^**b,c**^	**8.42 (4.43)**
Vitamin E (mg)	**15**	**6.74 (2.40)** ^**b,c**^	**5.49 (2.68)**
Folate-DFE (mcg)	**400**	**375.0 (126.1)** ^**a**^	**267.8 (117.5)**
Vitamin K (mcg)	120*	**110.3 (101.1)** ^**b,c**^	**88.2 (95.3)**
Pantothenic Acid (mg)	5*	22.3 (49.5)^b,c^	10.4 (33.0)
Calcium (mg)	**1200**	**1016.3 (373.8)** ^**b**^	**1031.4 (459.4)**
Copper (mg)	0.9	1.41 (0.88)^a^	1.08 (0.55)
Iron (mg)	9	13.6 (3.32)^b,c^	11.3 (3.17)
Magnesium (mg)	**420**	**315.1 (74.6)** ^**a**^	**265.4 (94.7)**
Manganese (mg)	2.3*	4.32 (1.38)^a^	2.98 (1.38)
Phosphorus (mg)	700	1465.9 (362.3)^a^	1355.8 (440.1)
Potassium (mg)	4700*	**3103.2 (768.3)** ^**a**^	**3111.3 (1035.6)**
Selenium (mcg)	55	116.3 (35.2)^a^	87.4 (44.5)
Sodium (mg)	2300*	3140.7 (830.2)^a^	2775.9 (920.3)
Zinc (mg)	**11**	**10.6 (3.22)** ^**b,c**^	**9.31 (3.88)**

An ANOVA was performed between the regular texture menus (n = 32) and the pureed texture menus (n = 32) among the 32 LTC homes from four provinces. Abbreviations: RDA = recommended dietary allowance; AI = adequate intake; M = male; SD = standard deviation; kcal = kilocalorie; g = gram; mg = milligram; mcg = microgram; RAE = retinol activity equivalent; NE = niacin equivalents; DFE = dietary folate equivalent. Bold text represents values under the RDA or AI

*Adequate Intake

^a^Significant interaction effect of province and texture between the two menu textures, i.e., there are differences at province and texture mean levels, but differences are not consistent p < 0.01

^b^Significant province effect i.e., differences occur among provinces in mean levels *p* < 0.01

^c^Significant texture effect, i.e., differences occur between textures in mean levels *p* < 0.01

In addition to fibre, nine micronutrients (vitamins B6, vitamin D, vitamin E, vitamin K, folate, calcium, magnesium, potassium, and zinc) in the regular texture and pureed menus *did not* meet the DRI recommendations when considering the average provided by homes in each of the four provinces. Pureed menus generally provided consistently lower values for nutrients, except vitamin D, calcium, and potassium when compared to the regular texture menu. Consistent with macronutrients and energy, 11 of the 22 micronutrients had a significant interaction effect between province and menu texture (*p* < 0.01), meaning that among provinces the micronutrient values were significantly different by province and there were also differences between menu textures; however the difference between regular and pureed was not consistent among provinces (i.e., differences were not consistently lower for pureed menus). These micronutrients were: vitamin B1, vitamin B3, vitamin B6, folate, copper, magnesium, manganese, phosphorus, potassium, selenium, and sodium (Table 3). A total of six micronutrients (vitamin D, vitamin E, vitamin K, pantothenic acid, iron, and zinc) of the 22 analyzed had no interaction but only main effects of both province and menu texture (*p* < 0.01) with the pureed menu texture consistently offering lower amounts of these micronutrients across all provinces. Additionally, vitamin A, vitamin B2, vitamin C, and calcium had a province only effect (*p* < 0.01) meaning that averages significantly varied by province but not by menu texture. Lastly, there were no significant main effects for vitamin B12 (*p* > 0.01) among provinces or between menu textures.

### Comparison of regular and pureed texture menus across homes by province

As with the cross-province comparison, variation existed in nutrient quality of menus by home and by menu texture (see Additional file 1: Tables S1-S4). Significant interaction effects for energy, some macronutrients and many micronutrients means that generalizations about pureed menus being poorer than regular texture menus with respect to nutritional quality could not be made globally. The pureed menu provided higher values for nutrients than the regular texture menu in Alberta (vitamin C, vitamin D, and calcium), Manitoba (protein, vitamin D, calcium, and potassium), New Brunswick (vitamin C), and in Ontario (protein, vitamin A, vitamin B2, vitamin B6, vitamin B12, vitamin D, calcium, copper, magnesium, phosphorous, potassium, sodium, and zinc). Alberta and Ontario had the fewest interaction effects. Further, Manitoba and Ontario had no menu texture only main effects, meaning that within LTC homes in these two provinces, pureed and regular textures were more likely to be consistent.

As compared to the national averages, homes in Alberta with regular texture menus were consistent with the national average with nine nutrients not meeting the DRI. Manitoba did better than the national average with only six nutrients in the regular texture and pureed menus not meeting the DRI. New Brunswick had menus that were least likely to meet the DRI for regular texture (11 nutrients) and pureed menus (15 nutrients). Ontario homes provided menus most likely to meet the DRI; six and five nutrients on the regular texture and pureed menus, respectively did not meet the DRI.

## Discussion

This study provides needed information on the energy, macronutrient, micronutrient, and fibre content for both regular texture and pureed texture LTC menus in Canada and provides implications for international audiences with respect to best practices for menu planning. This is the most comprehensive menu analysis of regular and pureed textures world-wide including 22 micronutrients among 32 LTC homes. Previous studies have investigated the pureed menu texture only [16], one or a few macronutrients [16, 17], or were based on a single LTC home menu [14, 15]. Using the average of four provinces, this study found that pureed menus typically provided lower amounts of nutrients compared to the regular texture menus. However, there were significant provincial and menu texture interactions for most comparisons, suggesting that overarching conclusions with respect to differences in nutritional quality could not be made by menu texture. New Brunswick had lower nutrient content for both menu textures as compared to other provinces, with Ontario having menus for both textures most likely to meet the DRI. Differences in menu planning and standards explain this variance.

There were no menus that met the DRIs for all macronutrients, micronutrients, and fibre in both food textures, suggesting that guidance and knowledge translation is still required to meet this standard. Fibre and nine micronutrients were below DRI recommendations for both menu textures among the provinces, indicating that greater attention is needed to emphasize ingredients and foods high in these nutrients. The capacity of homes within provinces to meet the DRI may be attributed to increased funds allocated for food [31]. To corroborate this contention, raw food cost was positively associated with energy provided by the pureed texture in this analysis. Ontario was the region most likely to meet the DRI and had the highest amount of dollars spent on raw food cost (mean = $8.50 per resident/day, range: $7.30–12.50). Not surprisingly, New Brunswick had the least amount of nutrients on average that met the RDA or AI and also had the lowest raw food expense (mean = $7.20 per resident/day, range: $6.30–7.90); this province does not have a raw food allocation from government funding, meaning that homes can shift food spending to other home priorities. This analysis also suggests some other best practices, such as shorter menu cycles (e.g., 3–4 weeks) and menus that are reviewed and revised more frequently; 7 of 8 Ontario homes had completed a full menu revision in the previous six months. Best practices suggested from this comparison include: per capita dollar allocation for food; sufficient funding for food; dietitian oversight to ensure menus meet the DRI; frequent revision of the menu (e.g. every 6 months); inclusion of family and residents in menu planning; and having a relatively short menu cycle (3–4 weeks) [6, 11].

Within each province, some homes had significantly lower nutrient content for pureed menus, while others did not, suggesting that quality menu planning goes beyond these provincial standards. Specifically in Ontario, there were fewer differences in micronutrients between pureed and regular texture menus, with some micronutrients actually higher in the pureed menu. A key difference in Ontario was the use of more standardized recipes that provided the same portion size to residents as the regular texture in this analysis; this necessitates providing more original product as volume decreases with texture modification (i.e., 275 ml when pureed becomes 200 ml), especially for pureed consistencies. This practice came about after intensive review at the provincial level, post research showing deficits in protein content of pureed offerings [10, 16]. Outside of New Brunswick, home differences appear to be more substantial than provincial differences. Homes with more frequent menu revision, larger and for-profit status were significantly and positively associated with increased values for protein and energy content. Facilities with these characteristics are providing more food or more nutrient dense products [31–33], which suggests that possibly more investment is being put into menu planning. Recipe development is also a key consideration, which varied by home within each province. Ontario and Manitoba homes were more likely to have standardized recipes for pureed foods and were more on par with regular menus for nutrient content. For example, vitamin D and calcium were on par for the regular and pureed menus. This finding could be explained by the addition of nutrients to pureed food during preparation (e.g., dairy products add energy, protein, calcium and vitamin D). Fortification of foods with infant cereal, skim milk powder, and powders containing both vitamins and minerals, or high energy and protein snacks has demonstrated improvements in nutrient density of foods [31–33]. Further best practices at the home level based on this analysis are that: standardized recipes for pureed and regular foods need to be developed and used consistently; nutrient analysis with comparison to the DRI needs to be completed for the full menu at the time of its development and for any substitutions; final pureed product portion size needs to be equivalent to the regular texture; nutrient intensification by choice of ingredients be used to promote better quality menus [18]; and food enhancement with easy to incorporate key nutrient dense products (e.g. skim milk powder) in pureed foods be a standard to promote nutrient density [33]. A repository of nutrient dense recipes that could be used by homes could also be developed and maintained by third party organizations such as food distributors.

These findings are consistent with prior work examining protein content of menus from LTC homes in Ontario and Saskatchewan, which found that not only did protein levels differ between LTC homes within each province but there were regional differences between the two provinces [16]. Furthermore, a study by Beck and Hanson assessed macronutrients of meal samples and found that the nutritional content varied among the 10 kitchens that participated in the study [17]. Contrary to some prior work [14–17] however was the finding in the current study that in some LTC homes the pureed texture menus provided the same or more nutrition than the regular texture menus. This demonstrates the importance of analyzing individual menus to truly understand and represent their content as menu practices are improving.

Commercial pureed food that is fortified or nutrient dense [31, 34, 35] may also be a solution for homes, especially when there are a small number of pureed consumers [26]. In these situations, commercial products may be more cost-effective than working up and preparing nutrient dense products in-house. The proportion of food production that was commercially prepared with no further preparation needed was lowest among LTC homes in Ontario, and is likely driven by the provincial standard that food be made in-house, including pureed products to promote consistency with regular offerings [10, 26]. Attention to nutrient density and portion size is evident in the higher number of homes in Ontario having pureed-specific recipes. When homes do not have standardized recipes for pureed menu items this leads to inconsistencies between the textures and possibly a lowered nutritional quality for pureed foods as quality would depend on the staff members (i.e., chefs) preparing the foods [16, 26]. The highest proportion of commercial products was in Manitoba, which provided the second most nutrient dense menus, with less variability between pureed and regular menus than Alberta and New Brunswick. The proportion of commercial food used was positively associated with energy provision from the regular texture menus among the 32 homes (Table 2) but not protein. Added calories are important, but micronutrients are also needed. A further best practice at the home level is that when in-home development and production of pureed foods is lacking, consider commercial products to fulfill this requirement of consistent nutrient dense products. Careful review of micronutrient content of any commercial product is needed prior to selection as quality varies by producer [36, 37] and sufficient energy does not necessarily equate to improved micronutrient profiles.

### Strengths and limitations

The cross-sectional design of this study does not allow for causal inferences; however, this is the first study to consider menu planning from different regions and a diverse set of homes, providing unique insight into factors worthy of further exploration and consideration. It is important to note that the eight homes included in this study for each province were purposefully sampled and had in many cases a prior relationship with research leads in each province. As such, these homes cannot be considered representative of their province on the key home characteristics described in this study (e.g. dietitian time). A limitation of this study was that not all LTC homes in each province provided menus that included snacks; therefore snacks were not included in this analysis. With the inclusion of snacks, the planned menus would have provided a higher amount of energy and macronutrients, and potentially micronutrients and fibre depending on the types of snacks offered. A recipe analysis for food based on ingredients using Food Processor software is a cost-effective substitute for a chemical analysis which is time-consuming and expensive to conduct [38]. A chemical analysis of duplicate portions is considered the gold standard to obtain accurate information on nutritional quality [38], and this has been done in previous research conducted on pureed foods in the LTC context [16]. However, nutrient analysis based on recipes comes with limitations, which include: accuracy of ingredients in recipes, accuracy of product labels, finding appropriate nutrient values for each ingredient, reliable and up to date nutrient composition data, converting units and household measures to weights, assigning weight change factors due to cooking the food, and errors in the nutrient analysis program based on recipe contents [37–39]. Further, not all commercial products, including pureed products provided a complete nutrient profile for all nutrients assessed in this study. Despite these limitations, a recipe analysis of LTC menus is an appropriate method because of the large sample size used in this study [28, 39]. Use of the same software database and code-book supported consistency across the four provinces and research assistants who analyzed the menus. A further limitation was that several of the pureed menus did not have standardized recipes on which to base the analysis and modifications to regular food recipes were required for these products. Although the analysts attempted to be methodical in how they calculated the potential nutrient content of these foods (for example using another home’s recipe for a similar product), there is the likelihood that this influenced findings. Misclassification may have also occurred for home level variables, as questions may not have been interpreted consistently among site management (e.g. raw food cost, dietitian time). Sites were contacted to confirm key variables for this analysis.

Despite these limitations, this study has several strengths. To date this is the most comprehensive and thorough menu analysis available, as a total of 32 LTC homes from four provinces provided their planned menus. Additionally, a variety of LTC homes were sampled. Research assistants were highly trained and steps were taken to ensure an accurate nutrient analysis of menus, recipes, and product labels. For example, research assistants consulted cooks or dietary food service managers to clarify items and portion sizes. Also, research assistants who worked together in each province checked each other’s nutrient analysis of items and the codebook in each province aided the research assistants to be consistent in database choices for menu items.

Future research should focus on consumption of both regular and modified texture foods, including pureed foods, since consumption is very different from provision. It is estimated that on average residents living in LTC consume about 50% of food offered [40, 41]. If planned menus are not meeting DRIs for all nutrients in addition to poor food intake, then residents in LTC will have nutritionally inadequate diets and possibly negative health consequences such as malnutrition [41–44]. Planned menus could also be examined with the inclusion of snacks to determine if the provision of snacks in addition to breakfast, lunch, and dinner meals would help menus reach recommended levels for nutrients. Lastly, future research should consider cost-effective menus that can provide nutrient-dense foods in LTC such as super-menus [18]. Research on strategies to support nutrient density is required with knowledge translation on how to improve menus.

## Conclusions

This study demonstrates the variability in menu planning in Canadian provinces and the need for improved menu planning protocols, including a complete nutrient analysis, to ensure planned diets meet nutrient requirements regardless of texture. In the Canadian context, menu planning is often regional or home specific and this study further illustrates differences due to inconsistent standards. Standardization of recipes for regular and pureed textures would promote consistency in nutrient content across LTC homes and regions; other best practices have also been identified with this analysis that can be transferred to other countries. With careful consideration of resident needs and deliberate menu planning, most vitamin and mineral needs can be met [8]. This study confirms prior research that suggests an iatrogenic effect of food provision to malnutrition in LTC. As noted by home and regional differences, when greater importance is placed on menu planning, potentially due to regulations, standards, and best practices this situation can be improved.

## Additional file

Additional file 1: Comparison of regular and pureed texture menus among the 32 homes by each province in the M3 study. **Table S1.** Comparison of menus among the eight LTC homes from Alberta. **Table S2.** Comparison of menus among the eight LTC homes from Manitoba. **Table S3.** Comparison of menus among the eight LTC homes from New Brunswick. **Table S4.** Comparison of menus among the eight LTC homes from Ontario. (DOCX 149 kb)

## Abbreviations

AB: Alberta
ANOVA: Analysis of variance
CFG: Canada’s Food Guide
DRI: Dietary Reference Intake
LTC: Long term care
M3: Making the Most of Mealtimes
MB: Manitoba
MTF: Modified texture food
NB: New Brunswick
ON: Ontario
RDA: Recommended dietary allowance
SD: Standard deviation
