# Supplementary material for: Nutritional quality of regular and pureed menus in Canadian long term care homes: an analysis of the Making the Most of Mealtimes (M3) project
Source: BMC Nutr. 2017 Oct 23;3:80. doi: 10.1186/s40795-017-0198-3 (PMC7050785; doi:10.1186/s40795-017-0198-3)
Supplement: Supplementary file 1 — Comparison of regular and pureed texture menus among the 32 homes by each province in the M3 study. Table S1. Comparison of menus among the eight LTC homes from Alberta. Table S2. Comparison of menus among the eight LTC homes from Manitoba. Table S3. Comparison of menus among the eight LTC homes from New Brunswick. Table S4. Comparison of menus among the eight LTC homes from Ontario. (DOCX 149 kb) [file 40795_2017_198_MOESM1_ESM.docx]

Supplementary Table 1. Comparison of menus among the eight LTC homes from Alberta.

| **Among LTC Homes in Alberta** | | | | | |
| --- | --- | --- | --- | --- | --- |
|  | **RDA (70+ M)**  **AI* (70+ M)** | **Regular Texture Menu** | | **Pureed Texture Menu** | |
|  |  | **Mean** | **SD** | **Mean** | **SD** |
| Energy (kcal) | n/a | 2066.8 ^a^ | 434.1 | 1720.2 | 382.5 |
| Protein (g) | 56 | 88.3 ^b,c^ | 23.6 | 78.4 | 16.9 |
| Carbohydrates (g) | 130 | 253.2 ^b,c^ | 47.9 | 209.8 | 47.1 |
| Fibre (g) | **30** | **22.5 ^b,c^** | **4.79** | **17.4** | **4.42** |
| Vitamin A (RAE) | **900** | 1056.6 | 793.6 | **810.2** | **465.0** |
| Vitamin B1 (mg) | 1.2 | 1.73 ^b,c^ | 0.59 | 1.31 | 0.57 |
| Vitamin B2 (mg) | 1.3 | 2.14 ^a^ | 0.83 | 2.13 | 0.92 |
| Vitamin B3-NE (mg) | 16 | 37.7 ^a^ | 12.4 | 27.2 | 11.0 |
| Vitamin B6 (mg) | **1.7** | 1.75 **^a^** | 0.70 | **1.32** | **0.65** |
| Vitamin B12 (mcg) | 2.4 | 5.18 | 5.11 | 4.61 | 2.12 |
| Vitamin C (mg) | 90 | 104.0 ^a^ | 50.0 | 111.9 | 51.3 |
| Vitamin D (mcg) | **20** | **5.18 ^a^** | **2.84** | **6.05** | **3.47** |
| Vitamin E (mg) | **15** | **7.26 ^a^** | **2.59** | **5.65** | **2.55** |
| Folate-DFE (mcg) | **400** | **332.0 ^a^** | **84.9** | **219.1** | **82.7** |
| Vitamin K (mcg) | **120 *** | **106.5 ^b,c^** | **78.5** | **69.6** | **84.4** |
| Pantothenic Acid (mg) | 5 * | 31.5 ^c^ | 57.1 | 8.7 | 14.7 |
| Calcium (mg) | **1200** | **849.5 ^a^** | **372.5** | **898.6** | **393.5** |
| Copper (mg) | 0.9 | 1.57 ^c^ | 1.08 | 0.97 | 0.25 |
| Iron (mg) | 9 | 13.7 ^b,c^ | 3.47 | 11.0 | 3.14 |
| Magnesium (mg) | **420** | **310.6 ^a^** | **82.8** | **237.1** | **67.9** |
| Manganese (mg) | 2.3 * | 4.49 ^b,c^ | 1.37 | 2.75 | 1.18 |
| Phosphorus (mg) | 700 | 1363.7 ^b,c^ | 389.8 | 1249.2 | 359.8 |
| Potassium (mg) | **4700 *** | **2887.1 ^a^** | **728.5** | **2783.1** | **746.7** |
| Selenium (mcg) | 55 | 118.5 ^a^ | 36.7 | 84.7 | 33.0 |
| Sodium (mg) | 2300 * | 3232.2 ^b,c^ | 953.3 | 2763.6 | 824.6 |
| Zinc (mg) | **11** | **10.9 ^b,c^** | **3.94** | **9.03** | **4.21** |

An ANOVA was performed between the regular texture menus (n= 8) and the pureed texture menus (n= 8) among the eight LTC homes in Alberta. Abbreviations: RDA= recommended dietary allowance; AI= adequate intake; M= male; SD= standard deviation; kcal= kilocalorie; g= gram; mg= milligram; mcg=microgram; RAE= retinol activity equivalent; NE= niacin equivalents; DFE= dietary folate equivalent. Bold text represents values under the RDA or AI.

* Adequate Intake.

^a^ Significant interaction effect of home and texture between the two menu textures, i.e., there are differences at home and texture mean levels, but differences are not consistent p<0.01.

^b^ Significant home effect i.e., differences occur among homes in mean levels p<0.01.

^c^ Significant texture effect, i.e., differences occur between textures in mean levels p<0.01.

Supplementary Table 2. Comparison of menus among the eight LTC homes from Manitoba.

| **Among LTC Homes in Manitoba** | | | | | |
| --- | --- | --- | --- | --- | --- |
|  | **RDA (70+ M)**  **AI* (70+ M)** | **Regular Texture Menu** | | **Pureed Texture Menu** | |
|  |  | **Mean** | **SD** | **Mean** | **SD** |
| Energy (kcal) | n/a | 2298.1 ^a^ | 359.1 | 2088.0 | 492.1 |
| Protein (g) | 56 | 90.9 ^b^ | 20.3 | 92.3 | 23.7 |
| Carbohydrates (g) | 130 | 304.3 ^a^ | 47.3 | 268.5 | 58.3 |
| Fibre (g) | **30** | **22.1 ^a^** | **5.16** | **17.0** | **6.41** |
| Vitamin A (RAE) | 900 | 1113.8 ^b^ | 418.4 | 1080.0 | 464.3 |
| Vitamin B1 (mg) | 1.2 | 1.85 ^b,c^ | 0.45 | 1.58 | 0.50 |
| Vitamin B2 (mg) | 1.3 | 3.23 ^a^ | 1.38 | 2.75 | 0.78 |
| Vitamin B3-NE (mg) | 16 | 34.8 ^a^ | 8.0 | 31.4 | 10.8 |
| Vitamin B6 (mg) | **1.7** | 1.74 ^b^ | 0.43 | **1.66** | **0.50** |
| Vitamin B12 (mcg) | 2.4 | 6.00 ^b^ | 1.91 | 5.96 | 1.80 |
| Vitamin C (mg) | 90 | 182.3 ^b^ | 97.4 | 163.6 | 88.4 |
| Vitamin D (mcg) | **20** | **9.37 ^b,c^** | **2.40** | **10.82** | **4.16** |
| Vitamin E (mg) | **15** | **7.66 ^a^** | **2.36** | **5.76** | **3.33** |
| Folate-DFE (mcg) | **400** | 465.9 ^a^ | 150.9 | **314.6** | **121.2** |
| Vitamin K (mcg) | **120 *** | **108.4 ^b^** | **63.3** | **84.0** | **55.3** |
| Pantothenic Acid (mg) | 5 * | 7.32 ^a^ | 1.91 | 7.23 | 2.23 |
| Calcium (mg) | 1200 | 1265.9 ^a^ | 325.3 | 1351.4 | 459.6 |
| Copper (mg) | 0.9 | 1.32 ^a^ | 0.32 | 1.17 | 0.40 |
| Iron (mg) | 9 | 15.2 ^b,c^ | 3.86 | 12.1 | 3.19 |
| Magnesium (mg) | **420** | **348.8 ^a^** | **72.4** | **314.3** | **96.0** |
| Manganese (mg) | 2.3 * | 4.53 ^a^ | 1.35 | 3.17 | 1.49 |
| Phosphorus (mg) | 700 | 1591.9 ^a^ | 353.3 | 1526.3 | 463.6 |
| Potassium (mg) | **4700 *** | **3380.6 ^b,c^** | **753.3** | **3687.9** | **1257.2** |
| Selenium (mcg) | 55 | 124.4 ^a^ | 42.0 | 94.2 | 51.6 |
| Sodium (mg) | 2300 * | 3357.4 ^a^ | 753.3 | 3094.7 | 1045.3 |
| Zinc (mg) | **11** | 11.5 ^a^ | 2.90 | **10.4** | **3.52** |

An ANOVA was performed between the regular texture menus (n= 8) and the pureed texture menus (n= 8) among the eight LTC homes in Manitoba. Abbreviations: RDA= recommended dietary allowance; AI= adequate intake; M= male; SD= standard deviation; kcal= kilocalorie; g= gram; mg= milligram; mcg=microgram; RAE= retinol activity equivalent; NE= niacin equivalents; DFE= dietary folate equivalent. Bold text represents values under the RDA or AI.

* Adequate Intake.

^a^ Significant interaction effect of home and texture between the two menu textures, i.e., there are differences at home and texture mean levels, but differences are not consistent p<0.01.

^b^ Significant home effect i.e., differences occur among homes in mean levels p<0.01.

^c^ Significant texture effect, i.e., differences occur between textures in mean levels p<0.01.

Supplementary Table 3. Comparison of menus among the eight LTC homes from New Brunswick.

| **Among LTC Homes in New Brunswick** | | | | | |
| --- | --- | --- | --- | --- | --- |
|  | **RDA (70+ M)**  **AI* (70+ M)** | **Regular Texture Menu** | | **Pureed Texture Menu** | |
|  |  | **Mean** | **SD** | **Mean** | **SD** |
| Energy (kcal) | n/a | 1727.9 ^a^ | 311.0 | 1286.6 | 347.3 |
| Protein (g) | 56 | 74.0 ^a^ | 15.8 | 61.5 | 17.1 |
| Carbohydrates (g) | 130 | 227.4 ^a^ | 44.8 | 164.3 | 54.0 |
| Fibre (g) | **30** | **18.4 ^b,c^** | **4.44** | **13.7** | **4.82** |
| Vitamin A (RAE) | 900 | 959.4 ^b^ | 775.8 | 901.4 | 639.4 |
| Vitamin B1 (mg) | **1.2** | 1.39 **^a^** | 0.39 | **0.94** | **0.53** |
| Vitamin B2 (mg) | 1.3 | 1.86 ^a^ | 0.71 | 1.64 | 1.08 |
| Vitamin B3-NE (mg) | 16 | 30.5 ^a^ | 7.26 | 20.9 | 10.8 |
| Vitamin B6 (mg) | **1.7** | **1.45 ^a^** | **0.34** | **1.08** | **0.49** |
| Vitamin B12 (mcg) | 2.4 | 5.43 ^b^ | 8.05 | 4.08 | 4.90 |
| Vitamin C (mg) | **90** | **83.7 ^a^** | **40.0** | 94.9 | 60.2 |
| Vitamin D (mcg) | **20** | **6.19 ^b^** | **4.24** | **6.06** | **3.52** |
| Vitamin E (mg) | **15** | **4.90 ^a^** | **1.58** | **4.08** | **1.65** |
| Folate-DFE (mcg) | **400** | **320.9 ^b,c^** | **114.6** | **188.1** | **99.9** |
| Vitamin K (mcg) | **120 *** | **68.1 ^b,c^** | **48.6** | **42.8** | **37.9** |
| Pantothenic Acid (mg) | **5 *** | 5.40 ^a^ | 1.47 | **4.68** | **1.68** |
| Calcium (mg) | **1200** | **796.0 ^a^** | **322.3** | **644.8** | **340.3** |
| Copper (mg) | **0.9** | 1.40 ^c^ | 1.34 | **0.80** | **0.86** |
| Iron (mg) | 9 | 11.8 ^a^ | 2.53 | 9.36 | 2.90 |
| Magnesium (mg) | **420** | **278.6 ^b,c^** | **71.3** | **183.4** | **78.4** |
| Manganese (mg) | **2.3 *** | 4.35 ^b,c^ | 1.56 | **2.28** | **1.37** |
| Phosphorus (mg) | 700 | 1298.6 ^b,c^ | 352.5 | 1001.3 | 324.4 |
| Potassium (mg) | **4700 *** | **2655.8 ^a^** | **654.3** | **2284.0** | **620.1** |
| Selenium (mcg) | 55 | 103.4 ^a^ | 28.8 | 56.8 | 32.5 |
| Sodium (mg) | **2300 *** | 2828.4 ^a^ | 769.6 | **2030.1** | **650.1** |
| Zinc (mg) | **11** | **9.0 ^a^** | **2.67** | **6.6** | **3.04** |

An ANOVA was performed between the regular texture menus (n= 8) and the pureed texture menus (n= 8) among the eight LTC homes in New Brunswick. Abbreviations: RDA= recommended dietary allowance; AI= adequate intake; M= male; SD= standard deviation; kcal= kilocalorie; g= gram; mg= milligram; mcg=microgram; RAE= retinol activity equivalent; NE= niacin equivalents; DFE= dietary folate equivalent. Bold text represents values under the RDA or AI.

* Adequate Intake.

^a^ Significant interaction effect of home and texture between the two menu textures, i.e., there are differences at home and texture mean levels, but differences are not consistent p<0.01.

^b^ Significant home effect i.e., differences occur among homes in mean levels p<0.01.

^c^ Significant texture effect, i.e., differences occur between textures in mean levels p<0.01.

Supplementary Table 4. Comparison of menus among the eight LTC homes from Ontario.

| **Among LTC Homes in Ontario** | | | | | |
| --- | --- | --- | --- | --- | --- |
|  | **RDA (70+ M)** | **Regular Texture Menu** | | **Pureed Texture Menu** | |
|  | **AI* (70+ M)** | **Mean** | **SD** | **Mean** | **SD** |
| Energy (kcal) | n/a | 2140.7 ^a^ | 220.6 | 2108.6 | 278.6 |
| Protein (g) | 56 | 92.8 ^a^ | 17.3 | 96.4 | 18.8 |
| Carbohydrates (g) | 130 | 278.4 ^a^ | 34.3 | 274.1 | 36.6 |
| Fibre (g) | **30** | **20.5 ^b^** | **4.89** | **19.6** | **5.31** |
| Vitamin A (RAE) | 900 | 1117.5 ^b^ | 353.6 | 1138.0 | 343.0 |
| Vitamin B1 (mg) | 1.2 | 1.71 ^b^ | 0.44 | 1.71 | 0.51 |
| Vitamin B2 (mg) | 1.3 | 2.50 ^a^ | 0.40 | 2.59 | 0.44 |
| Vitamin B3-NE (mg) | 16 | 36.2 ^b^ | 9.79 | 35.7 | 11.6 |
| Vitamin B6 (mg) | 1.7 | 1.80 ^b^ | 0.48 | 1.84 | 0.56 |
| Vitamin B12 (mcg) | 2.4 | 5.61 ^a^ | 1.53 | 5.89 | 1.76 |
| Vitamin C (mg) | 90 | 152.4 ^b^ | 49.4 | 144.2 | 48.2 |
| Vitamin D (mcg) | **20** | **9.34 ^b^** | **3.45** | **10.8** | **3.87** |
| Vitamin E (mg) | **15** | **7.15 ^b^** | **1.96** | **6.48** | **2.41** |
| Folate-DFE (mcg) | **400** | **381.3 ^b^** | **89.8** | **349.4** | **80.9** |
| Vitamin K (mcg) | 120 * | 158.1 | 157.3 | 156.5 | 134.3 |
| Pantothenic Acid (mg) | 5 * | 45.1 | 74.3 | 20.9 | 63.5 |
| Calcium (mg) | **1200** | **1154.0 ^a^** | **239.9** | 1231.0 | 242.5 |
| Copper (mg) | 0.9 | 1.34 ^b^ | 0.28 | 1.37 | 0.27 |
| Iron (mg) | 9 | 13.7 ^b^ | 2.39 | 12.9 | 2.29 |
| Magnesium (mg) | 420 | 322.2 ^b^ | 53.0 | 326.6 | 50.1 |
| Manganese (mg) | 2.3 * | 3.91 ^a^ | 1.18 | 3.72 | 1.06 |
| Phosphorus (mg) | 700 | 1609.4 ^a^ | 234.1 | 1646.2 | 284.5 |
| Potassium (mg) | **4700 *** | **3489.3 ^a^** | **487.8** | **3690.0** | **583.0** |
| Selenium (mcg) | 55 | 118.7 ^a^ | 28.7 | 113.9 | 38.8 |
| Sodium (mg) | 2300 * | 3144.9 ^b^ | 755.2 | 3215.2 | 610.3 |
| Zinc (mg) | 11 | 11.2 ^a^ | 2.63 | 11.2 | 2.97 |

An ANOVA was performed between the regular texture menus (n= 8) and the pureed texture menus (n= 8) among the eight LTC homes in Ontario. Abbreviations: RDA= recommended dietary allowance; AI= adequate intake; M= male; SD= standard deviation; kcal= kilocalorie; g= gram; mg= milligram; mcg= microgram; RAE= retinol activity equivalent; NE= niacin equivalents; DFE= dietary folate equivalent. Bold text represents values under the RDA or AI.

* Adequate Intake.

^a^ Significant interaction effect of home and texture between the two menu textures, i.e., there are differences at home and texture mean levels, but differences are not consistent p<0.01.

^b^ Significant home effect i.e., differences occur among homes in mean levels p<0.01.

^c^ Significant texture effect, i.e., differences occur between textures in mean levels p<0.01.
